# Controlled knowledge updating in memory-enabled AI agents: beyond model editing and recall

**DOI:** 10.3389/fdata.2026.1938137

**Published:** 2026-09-28

**Authors:** Gabriel Chavira Juárez, Eder Jahir Gonzalez Bravo, Guadalupe Esmeralda Rivera García, Javier A. Arcos Espinosa, Salvador W. Nava-Díaz

**Affiliations:** 1 Facultad de Ingeniería Tampico, Universidad Autónoma de Tamaulipas, Tampico, Tamaulipas, Mexico; 2 Tecnológico Nacional de México/Instituto Tecnológico Superior de Pánuco, Pánuco, Veracruz, Mexico

**Keywords:** agent evolution, agent memory, benchmarking, controlled knowledge updating, knowledge editing, LLM agents

## Abstract

Large language model agents that persist across sessions, tools, users, and changing environments do more than answer isolated prompts; they accumulate state. When new evidence arrives, the central question is where that change should live: transient context, external memory, tool or workflow definitions, activation states, or model parameters. Recent benchmarks compare update mechanisms and assess memory over time, while emerging architectures coordinate multiple memory types. In this Perspective, we examine how a decision layer should select among update substrates and evaluate whether its choices remain appropriate. A controlled update is not merely a successful edit or a recalled fact; it is a decision to alter the least invasive substrate sufficient for the claim's scope, expected persistence, and evidentiary strength, including when the correct action is not to write persistent state. We propose a substrate-aware view of controlled knowledge updating organized by three principles: substrate proportionality, temporal defeasibility, and auditable continuity. This framing treats knowledge updating as a longitudinal control problem and motivates evaluation criteria that include update selection, temporal consistency, interference, reversibility, efficiency, and robustness. The resulting agenda connects memory, knowledge editing, agent evolution, and benchmarking under a single practical question: how should an agent decide what to change so that future behavior improves without accumulating uncontrolled state?

## 1 Introduction

Large language model (LLM) agents are moving from single-turn text generation toward systems that plan, call tools, reflect on feedback, and maintain state across extended interactions (Wang L et al., 2024; Yao et al., 2023; Schick et al., 2023; Qin et al., 2024; Patil et al., 2024; Liu X et al., 2024). This transition changes the meaning of knowledge updating. In a stateless model, updating knowledge often means retraining, fine-tuning, or targeted model editing. For memory-enabled agents, future behavior can also be changed through retrieved evidence, external memory, reflective notes, edited parameters, steering vectors, tool libraries, workflow changes, or explicit forgetting. In data-intensive deployments, this substrate choice is not merely an implementation detail: stale documents, user traces, tool outputs, and revised assumptions can continue to shape behavior after their validity has changed.

Recent work has advanced three pieces of this picture. Agent memory and reflection systems show how agents can store experience, synthesize reflections, update memories, and forget selectively (Park et al., 2023; Zhong et al., 2024; Shinn et al., 2023). More recent architectures move beyond passive storage toward active memory organization: A-Mem dynamically links and revises memory representations as experience accumulates, while MemoryOS coordinates storage, updating, retrieval, and generation across hierarchical memory levels (Xu et al., 2025; Kang et al., 2025). Recurrence, retrieval, and explicit memory mechanisms expand usable context and access to evidence (Dai et al., 2019; Wu et al., 2022; Borgeaud et al., 2022). Model-editing research has progressed from learned editors and memory-based editors to localization, mass editing, and evaluation frameworks (De Cao et al., 2021; Mitchell et al., 2022a,b; Meng et al., 2022, 2023; Wang P et al., 2024; Li et al., 2024). In parallel, LoCoMo exposes difficulties in long-term memory and temporal reasoning, while AgentBench, WebShop, Mind2Web, WebArena, API-Bank, and SWE-bench evaluate interactive agency, web navigation, tool use, and software tasks (Maharana et al., 2024; Liu X et al., 2024; Yao et al., 2022; Deng et al., 2023; Zhou et al., 2024; Li M et al., 2023; Jimenez et al., 2024).

WikiBigEdit compares knowledge editing with retrieval augmentation and continual fine-tuning under sequential factual updates, assessing retention, generalization, and interference (Thede et al., 2025). MemoryAgentBench includes test-time learning, long-range understanding, and selective forgetting alongside accurate retrieval, comparing long-context, retrieval-based, and agentic memory systems (Hu et al., 2026). The MemOS pre-print describes an architecture that coordinates plaintext, activation-based, and parameter-level memory (Li et al., 2025). Its scheduler considers context and stability, and its lifecycle management is supported by provenance, versioning, and access controls.

Our contribution is an integrative framework for judging when a particular update mechanism is justified. It relates substrate choice to the evidence supporting a claim, its intended scope, and expected persistence, including cases where no persistent write is appropriate. These criteria guide evaluation across architectures, with separate assessment of selection, execution, and maintenance.

## 2 Beyond editing and recall

Knowledge editing has clarified an important goal: a model should incorporate a targeted update while preserving unrelated knowledge (Wang et al., 2025). This goal is essential when the update is global, stable, and should affect many future contexts. Here, “global” means system-wide within the agentic deployment considered, rather than specific to a user, task, or session. However, memory-enabled agents often face updates that vary in scope. A user's temporary preference, a project-specific constraint, a tool failure, a contradicted observation, or a revised plan may not justify parametric editing. Conversely, storing every observation in memory can create stale, conflicting, or privacy-sensitive state that later contaminates reasoning. At the parameter level, this risk resembles catastrophic interference in continual learning, where new learning can degrade previously learned behavior (Kirkpatrick et al., 2017).

Recall is also insufficient as the dominant memory objective. Long-term memory and long-context benchmarks show that agents struggle with multi-session reasoning, temporal change, positional sensitivity, and knowledge updates (Maharana et al., 2024; Liu N et al., 2024; Bai et al., 2024). Retrieval itself must be conditional: REALM, DPR, and RAG improved retrieval and retrieval-augmented generation; Self-RAG adds self-critique; and RAGAs provides evaluation methods for RAG pipelines (Guu et al., 2020; Karpukhin et al., 2020; Lewis et al., 2020; Asai et al., 2024; Es et al., 2024). These approaches do not, by themselves, settle when retrieved evidence should become persistent agent state. More recall is therefore not automatically better. A reliable agent must remember current information, reduce the influence of obsolete information, and treat absent evidence as a reason for abstention rather than fabrication.

The issue becomes sharper for agent evolution. Surveys and taxonomies of LLM knowledge mechanisms increasingly treat knowledge as something that is acquired, used, and revised over time (Wang M et al., 2024). AgeMem further treats storing, retrieving, updating, summarizing, and discarding memory as tool-based actions learned within a unified agent policy (Yu et al., 2026). This advances update selection within the memory subsystem; the broader question addressed here is how an agent should choose across memory and other behavior-shaping substrates. For agents, evolution is not only improvement of a model; it is the accumulation of persistent changes across memory, prompts, tools, policies, traces, and learned routines. Without control, this accumulation becomes technical debt: the agent may behave as if outdated information were still valid, overgeneralize a local correction, or hide the provenance of a consequential update.

## 3 Substrate-aware updating

We define a knowledge update as any intentional change that alters an agent's future behavior in light of new evidence. Controlled knowledge updating is the practice of selecting the least invasive substrate sufficient for the intended scope, including the option not to write persistent state, applying the change with provenance, and evaluating its downstream effects over time. The central question shifts from “can the system update?” to “what should change, and how can we know that the change remains appropriate?”

We use *substrate* to mean a component of the agentic system whose content, state, or configuration can be changed to shape subsequent behavior, whether within an interaction or across sessions. Examples include context, external memory, model parameters, activations, and reusable tool or workflow definitions. They are comparable as intervention targets in terms of scope, persistence, reversibility, and auditability. An update mechanism acts on a substrate: model editing changes parameters, while forgetting can remove or attenuate information across several substrates.

Three principles make this perspective operational. *Substrate proportionality* means that update persistence and blast radius should match the scope of the claim, the confidence of the evidence, and the expected half-life of the information. *Temporal defeasibility* means that persistent knowledge remains revisable: each update should carry validity conditions and a path for revision, attenuation, or rollback. *Auditable continuity* means that future behavior remains traceable to update decisions, sources, and affected substrates. Together, these principles translate the “least invasive substrate” into concrete decision variables: scope, evidence confidence, expected duration, reversibility requirement, interference tolerance, and audit requirement. Here, invasiveness means the combined blast radius, difficulty of reversal, opacity of audit trails, and computational cost of a change.

Figure 1 summarizes the proposed control loop. The loop begins with an update candidate: a user correction, environmental feedback, new document, contradiction, failed action, or instruction. The agent first classifies the evidence and intended scope. Uncertain or transient claims may remain in retrieval or context injection. User-specific or task-specific claims may move to external memory. Stable global corrections may justify a curated global update, such as an external knowledge-base change or, under stricter evidence and regression tests, model editing. Behavioral modulation may be better handled through steering (Li K et al., 2023); changes in how the agent acts may belong in tools or workflows; obsolete, harmful, or unauthorized information may call for forgetting or attenuation. A reference policy can remain qualitative while making thresholds explicit: transient low-confidence evidence stays in context, repeated user-level evidence moves to memory, and stable high-confidence global corrections meet stronger evidentiary thresholds before model editing is attempted. Learning or standardizing such policies is part of the research agenda.

**Figure 1:**
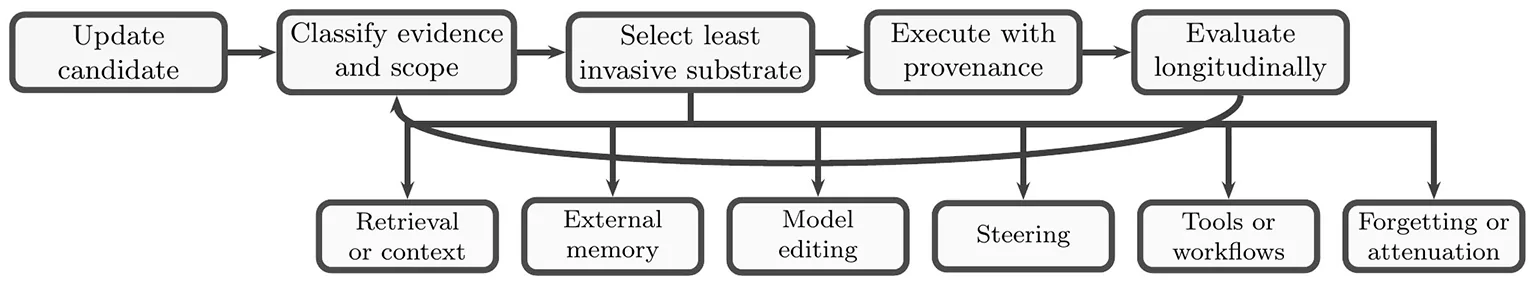
Controlled knowledge updating as a substrate-selection loop. The core decision is which substrate should change for a given evidence source, scope, persistence requirement, and risk profile. The branches indicate intervention options, including mechanisms acting on the selected substrate; forgetting or attenuation can apply across substrates.

Updating a tool or workflow means changing a reusable procedure in response to evidence, such as correcting a workflow after a recurring failure. Querying a tool supplies information; it does not by itself update the tool or establish the authority of its output. The scope here is bounded runtime adaptation: proposed changes require supporting evidence, an explicit scope, validation before reuse, and a revision or rollback path.

Consider, for instance, an assistant serving the same user across sessions. A one-off “keep it short today” would classify as transient evidence with no persistent effect; the same preference confirmed across several unrelated sessions would move to external memory, since evidence has accumulated and the scope is user-level; a later request for full detail on a single report would remain a same-turn override, leaving the stored preference untouched; and an unverified instruction embedded in a retrieved document would be logged and refused rather than written to any substrate. Structurally similar “the user said X” inputs are thus routed to different substrates once scope, evidence accumulation, and persistence are taken into account.

Forgetting becomes part of the same control problem. Rather than a memory failure alone, it is a control action that limits the future influence of obsolete, contradicted, unsafe, or low-value state. MemoryBank already illustrates selective preservation and forgetting as part of long-term interactions (Zhong et al., 2024). A recent graph-based perspective on affective conversational agents similarly models persistent memory through reinforcement, decay, pruning, and structural reorganization, illustrating that forgetting may alter relationships within memory rather than merely delete stored content (Albanese, 2026). Forgetting can mean deletion, decay, masking, reduced retrieval priority, provenance-preserving archival, or replacement by a superseding memory; these choices have different privacy, reliability, and audit consequences. The broader implication is that forgetting belongs alongside recall in evaluation, because a system that remembers everything can still be unreliable if it cannot attenuate the influence of what should no longer guide behavior.

Hard cases make the control problem visible. Supersession occurs when a once-valid memory is replaced by newer evidence; contradiction occurs when two retained items cannot both guide behavior; personalization drift occurs when a local preference is overgeneralized across contexts; privacy revocation occurs when previously authorized information should no longer be used; and adversarial memory injection occurs when an external actor tries to persist a malicious instruction or false fact through an integrated application or poisoned retrieval corpus (Greshake et al., 2023; Zhong et al., 2023). Privacy revocation in runtime memory is distinct from training-data machine unlearning, but both problems share the goal of limiting the future influence of information that should no longer affect behavior (Cao and Yang, 2015; Bourtoule et al., 2021). Failure to separate these cases reflects weak governance of persistent state.

## 4 Evaluation as longitudinal control

Evaluation of substrate choice complements the method comparisons and memory architectures discussed above (Thede et al., 2025; Hu et al., 2026; Li et al., 2025). A correct response does not establish that the underlying update was justified. An agent may, for example, answer correctly after storing information that should have remained temporary. Table 1 outlines criteria for assessing whether an agent controls knowledge updates rather than merely accumulating state.

**Table 1:** Evaluation criteria for controlled knowledge updating.

Criterion and error class	Operational test	Failure signal
Scope fit (selection)	Does the selected substrate match the scope, expected duration, and evidence strength of the update?	A local preference becomes a global behavioral change, or a stable correction remains trapped in transient context.
Evidence quality (selection)	Does the agent update, defer, or refuse according to source reliability and uncertainty?	The agent persists rumors, adversarial instructions, or single-instance noise.
Temporal validity (maintenance)	Do future probes distinguish current, superseded, and uncertain information?	Obsolete memories continue to influence future reasoning.
Interference (execution)	Does the update preserve unrelated capabilities, facts, and memories?	A correction improves one case while degrading adjacent behavior.
Reversibility (maintenance)	Can the update be rolled back, attenuated, or superseded when evidence changes?	The system accumulates irreversible state from weak evidence.
Auditability (execution)	Can the system report what changed, why, from what source, and in which substrate?	Future behavior cannot be traced back to update decisions.
Efficiency (selection)	Does the agent choose a cost-appropriate mechanism when a lighter substrate would suffice?	Expensive editing or retrieval is used where a simpler update would work.
Robustness (maintenance)	Does the update remain reliable under contradiction, missing evidence, or adversarial memory?	The agent amplifies poisoned, inconsistent, or fabricated state.

These criteria complement existing measures of memory effectiveness, knowledge-editing reliability, and agent task success.

Benchmarks should therefore test more than whether an agent can answer a question from prior sessions. They should include cases where new information supersedes old information, where evidence is ambiguous, where the correct action is not to update, and where the same information belongs in different substrates depending on scope. For example, “the user prefers short answers today” should not be treated like “the capital of a country has changed”; a temporary preference is usually contextual or memory-scoped, while a stable public fact may justify broader updating.

A minimal benchmark unit would contain four phases. First, an update event presents new evidence together with uncertainty, source, scope, and expected duration. Second, the agent must choose a substrate or refuse to update, with a short justification. Third, the system is immediately probed for factual correctness and interference. Fourth, delayed probes test temporal consistency, supersession, abstention, and audit trace. Selection can be scored against a reference policy or expert labels, while execution and maintenance can be scored by behavioral probes. Scoring can then separate selection error, execution error, and maintenance error rather than reducing all failures to a single end-task score.

Since substrate selection involves policy judgment, evaluation should allow a set of admissible choices rather than a single gold label. A benchmark instance can be annotated by multiple experts who assign an update event to one or more acceptable substrates and record the rationale in terms of scope, evidence strength, expected duration, reversibility, and audit needs. Disagreement should be reported rather than hidden: high-agreement cases test basic competence, while low-agreement cases identify ambiguous update policies and can be scored with partial credit or preference rankings. The primary unit of analysis should be the update event, with scores aggregated over trajectories to capture accumulation, supersession, and interference across time.

The distinction also matters for steering and model editing. Steering can adjust behavior without parameter changes, while model editing can alter factual associations more directly (Meng et al., 2022, 2023). Controlled updating evaluates when each mechanism is warranted. A strong system sometimes refuses to edit, sometimes stores a memory, sometimes retrieves evidence, and sometimes forgets. Its competence lies in this selection behavior.

## 5 Research agenda

The first priority is update triage: agents need policies that classify evidence, scope, persistence, and risk before changing state. These policies could be rule-based, learned, or hybrid, but they should be explicit enough to audit. These assessments provide the basis for substrate selection.

Memory design is a second priority, with provenance and validity tracked across other substrates as well. Memory items should retain their source, time, confidence, validity conditions, and supersession status. Corresponding records should link parameter edits, steering configurations, and workflow revisions to their evidence and justification; they can be maintained outside the modified substrate. These records identify updates that need revision or rollback as evidence expires or is superseded. They support longitudinal maintenance and assessment of auditability and temporal validity (Table 1).

Cross-substrate benchmarking is a third priority, building on the selection criteria established by triage policies and the update traces provided by provenance records. Benchmarks of long-term memory and agent task performance (Maharana et al., 2024; Liu X et al., 2024) could be extended with controlled update trajectories. These would assess selection, execution, and maintenance under delayed contradiction or supersession using the criteria in Table 1.

Finally, forgetting should be an explicit design and evaluation target within longitudinal maintenance. The relevant question is not only whether information disappears from recall, but which mechanism limits its future influence and whether that mechanism preserves the required audit trail. Treating deletion, decay, masking, archival, and supersession as interchangeable would repeat the same substrate confusion that currently affects updating.

## 6 Discussion

The central design issue is no longer whether an agent can store more interaction history or apply an isolated factual correction. It is whether the system can keep future behavior aligned with the evidence, scope, and duration of each update. Model editing, retrieval, steering, and memory operations act on different substrates. The decision layer should record why a substrate was chosen so that the choice can be audited and reconsidered when the evidence changes.

Several boundary conditions follow from this view. Substrate selection is not a universal mapping from evidence type to mechanism; it depends on domain risk, privacy constraints, deployment policy, computational cost, and the acceptable level of expert disagreement. The goal is therefore not to prescribe a single substrate for every update, but to make the selection policy explicit enough to audit, compare, and revise.

Our unit of analysis is an agentic deployment governed by a defined update policy. A global update is intended to apply across relevant future contexts within that deployment. Modifying a shared knowledge base or model may also affect other deployments, so these potential effects must be considered before selecting a substrate. Cross-system updates require authority over the shared resource, provenance identifying affected systems, conflict resolution, and coordinated rollback. A protocol for coordination across independently managed systems remains outside the present scope.

The practical value of controlled knowledge updating is restraint. A well-designed agent avoids parametric edits for local or uncertain information, persistent memory for transient context, reliance on superseded evidence as if it were current, and irreversible deletion when traceability is required. The research challenge is to make these decisions measurable. The long-term aim is not agents that remember more, but agents that know what deserves to persist, what should remain provisional, and what must be revised, attenuated, or forgotten.

## Data Availability

The original contributions presented in the study are included in the article/supplementary material, further inquiries can be directed to the corresponding author.
